# Supplementary material for: A Reduced Starch Level in Plants at Early Stages of Infection by Viruses Can Be Considered a Broad-Range Indicator of Virus Presence
Source: Viruses. 2022 May 28;14(6):1176. doi: 10.3390/v14061176 (PMC9227243; doi:10.3390/v14061176)
Supplement: Supplementary file 1 [file viruses-14-01176-s001.zip › viruses-1720472-supplementary.pdf]

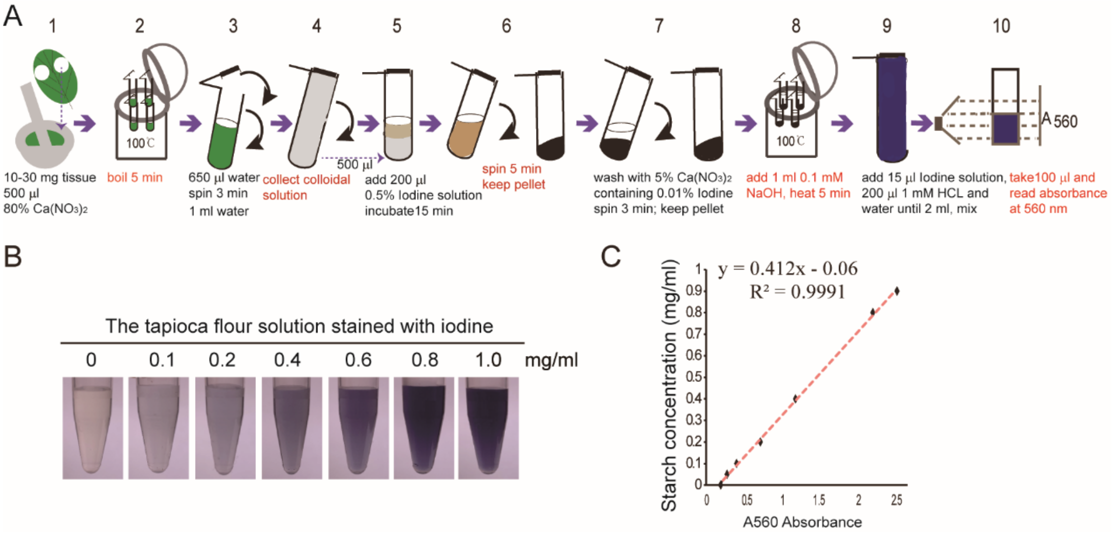

Figure S1. Purification and determination of starch content in leaves. **(A)** A schematic diagram detailing the procedure for the quantification of starch content in leaves. (B) The series of concentration of tapioca flour solutions (0, 0.1, 0.2, 0.4, 0.6, 0.8, 1.0 mg/ml water) were stained with iodine described in (A). **(C)** Calibration graph of absorbance at 560 nm against concentration (mg/ml) of tapioca flour solutions described in (B), the linear regression equation Y (starch concentration, mg/ml) = 0.412X (absorbance) − 0.06 (correlation coefficient, R^2 = 0.9991).

Table S1. Primers were used in this study.

Primer Name	Oligonucleotide sequence (5'- 3')	GenBank Accession Number	Size of PCR product
F-CMV-RNA3	TCCCTTGCCGAAATTCGATTCTAC	MG025949	
R-CMV-RNA3	CGTACCCTGAAACTAGCACGTTG	MG025949	589 bp
F-TMV	TGCTGCATGCCCAACCTGGACGTTC	AF165190	
R-TMV	AGACTTCCTTGCGCGTCCTCTTACT	AF165190	805 bp
F-PVX	ATTGCCGATCTCAAGCCACT	NC_011620.1	
R-PVX	GGGGTAGGCGTCGGTTAT	NC_011620.1	800 bp
F-PVY-CP	ATGCAAGCAAATGACACAATCGATG	JF927760.1	
R-PVY-CP	TCACATGTTCTTAACTCCAAGTAG	JF927760.1	810 bp
F-AGV	CCTTCTGGACAAGAGCAA	KM386645	
R-AGV	AGATGATTGGGTGAGTGT	KM386645	496 bp
F-ASGV-CP	CCCGCTGTTGGATTTGATACACCTC	NC_001749.2	
R-ASGV-CP	CTGCAAGACCGCGACCAAGTTT	NC_001749.2	524 bp
F-ASPV-CP	ATGTCTGGAACCTCATGCTGCAA	NC_003462.2	
R-ASPV-CP	TTGGGATCAACTTTACTAAAAAGCATAA	NC_003462.2	363 bp
F-ACLSV-CP	TCTGCAAGAGAATTTCAGTT	KC935956.1	
R-ACLSV-CP	GTCTACAGGCTATTTATTATAAG	KC935956.1	824 bp
F-Nb18S	GCAAGACCGAAACTCAAAGG	TC23401	
R-Nb18S	TGTTCATATGTCAAGGGCTGG	TC23401	150 bp
F-MdEF-1α	ATTCAAGTATGCCTGGGTGC	NC_041799	
R-MdEF-1α	CAGTCAGCCTGTGATGTTCC	NC_041799	174 bp
F-NbAGPase L1-RT	TGCTACAGGCTAATAGACATCCC	NbS00018336g0015.1	
R-NbAGPase L1-RT	ACCTCAACAAATCCGTCTCCAAA	NbS00018336g0015.1	158 bp
F-NbAGPase L2-RT	CCGTTCTACACTTCTCCT	NbS00011082g0009.1	
R-NbAGPase L2-RT	ATAGTCCGCACCCATCAT	NbS00011082g0009.1	186 bp
F-NbAGPase L3-RT	ATGATGCTTGGTGCTGACTACTATG	NbS00050736g0008.1	
R-NbAGPase L3-RT	TCTGCTTCTTGTACGCCCTCTG	NbS00050736g0008.1	179 bp
F-NbAGPase S-RT	CCACACACTCCATAGCAGA	Niben101Scf01625	
R-NbAGPase S-RT	AGAGATGAGACGACGAAAA	Niben101Scf01625	174 bp
F-NbGBSS-RT	CACAATGGGTTGAGGGC	Niben101Scf08819	
R-NbGBSS-RT	CAGGGCTGGTGGTAGTC	Niben101Scf08819	231 bp
F-NbGWD-RT	GATGAAGGTCCAAAGCG	Nbv6.1trP17673	
R-NbGWD-RT	GACAGCCATGCACAGAT	Nbv6.1trP17673	132 bp
